# SINHCAF/FAM60A and SIN3A specifically repress HIF-2α expression

**DOI:** 10.1042/BCJ20170945

**Published:** 2018-06-29

**Authors:** John Biddlestone, Michael Batie, Daniel Bandarra, Ivan Munoz, Sonia Rocha

**Affiliations:** 1 Centre for Gene Regulation and Expression, School of Life Sciences, University of Dundee, Dundee DD1 5EH, U.K.; 2 SCREDS Clinical Lecturer in Plastic and Reconstructive Surgery, Centre for Cell Engineering, University of Glasgow, Glasgow G12 8QQ, U.K.; 3 Department of Biochemistry, Institute for Integrative Biology, University of Liverpool, Liverpool L69 7ZB, U.K.; 4 MRC Protein Phosphorylation Unit, School of Life Sciences, University of Dundee, Dundee DD1 5EH, U.K.

**Keywords:** HIF-2histone deacetylases, hypoxia, hypoxia-inducible factors, SIN3A, SP1, transcription

## Abstract

The SIN3A–HDAC (histone deacetylase) complex is a master transcriptional repressor, required for development but often deregulated in disease. Here, we report that the recently identified new component of this complex, SINHCAF (SIN3A and HDAC-associated factor)/FAM60A (family of homology 60A), links the SIN3A–HDAC co-repressor complex function to the hypoxia response. We show that SINHCAF specifically represses HIF-2α mRNA and protein expression, via its interaction with the transcription factor SP1 (specificity protein 1) and recruitment of HDAC1 to the HIF-2α promoter. SINHCAF control over HIF-2α results in functional cellular changes in *in vitro* angiogenesis and viability. Our analysis reveals an unexpected link between SINHCAF and the regulation of the hypoxia response.

## Introduction

SINHCAF [SIN3A and HDAC (histone deacetylase)-associated factor, HUGO nomenclature]/FAM60A (family of homology 60A) is a poorly characterized protein that was originally shown to be part of the SIN3A repressor complex by two independent studies [1,2]. SIN3A is a multi-protein complex with function in developmental processes such as stem cell function [3] but also in pathological processes such as cancer [4]. It controls cellular metabolism, cell cycle, and cell survival [4]. Despite limited knowledge on SINHCAF function, a recent study identified a single-nucleotide polymorphism present on SINHCAF which is associated with type II diabetes in a cohort of Japanese patients [5]. SINHCAF has been associated with eosophageal cancer, by an integrative analysis of copy number [6], and more recently shown to control SIN3A loading in many important genes for stem cell maintenance in mouse embryonic stem cells [7].

Solid tumors are often characterized by the presence of low oxygen or hypoxia [8]. Adaptation and survival to such environments is mediated by a family of transcription factors called hypoxia-inducible factors (HIFs). Transcriptional target genes that are controlled by the HIFs code for proteins that are involved in important cellular processes including energy homeostasis, migration, and differentiation [9–11]. Deregulation of the HIF system has been shown to be important in the development of multiple disease processes including cancer progression and stem cell differentiation [12,13].

HIF is a heterodimeric transcription factor that consists of a constitutively expressed HIF-1β subunit and an O_2_-regulated HIF-α subunit [14]. Three isoforms of HIF-α have been identified (HIF-1α, -2α, and -3α). The HIF-α isoforms are all characterized by the presence of bHLH (basic helix–loop–helix)–PAS [Per/ARNT (aryl hydrocarbon nuclear translocator)/Sim] and ODD (oxygen-dependent degradation) domains. Both HIF-1α and HIF-2α have important cellular functions as transcription factors with some redundancy in their targets [15,16]. HIF-2α protein shares sequence similarity and functional overlap with HIF-1α, but its distribution is restricted to certain cell types, and in some cases, it mediates distinct biological functions [17].

The regulation of the HIF-α subunits is best understood at the post-transcriptional level and is mediated by hydroxylation-dependent proteasomal degradation. In well-oxygenated cells, HIF-α is hydroxylated in its ODD. This proline hydroxylation is catalyzed by a class of dioxygenase enzymes called prolyl hydroxylases (PHDs). PHDs require Fe^2+^ and α-ketoglutarate as cofactors for their catalytic activity, and have an absolute requirement for molecular oxygen as a co-substrate, making their activity reduced in hypoxia [18–21]. Prolyl-hydroxylation of HIF-α attracts the von Hippel–Lindau (vHL) tumor suppressor protein, which recruits the Elongin C–Elongin B–Cullin 2–E3–ubiquitin–ligase complex, leading to the Lys48-linked polyubiquitination and proteasomal degradation of HIF-α [22–24]. In hypoxia, HIF-α is stabilized, can form a heterodimer with HIF-1β in the nucleus, and bind to the consensus *cis*-acting hypoxia-response element (HRE) nucleotide sequence 5′-RCGTG-3′, which is present within the enhancers and/or promoters of HIF target genes [25–27]. HIF-α stabilization therefore allows the cell to enact a transcriptional program that is appropriate to the hypoxic environment [28].

In contrast with the post-translation regulation of HIF, the factors that regulate the basal expression of the HIF-α isoforms are only now being investigated. Deregulation of HIF basal expression has been linked to the development of solid tumors [29]. The transcriptional regulator nuclear factor kappaB (NF-κB) is a direct modulator of HIF-1α expression, in basal and hypoxic conditions, as well as in response to inflammatory stimulus [21,30–33]. NF-κB also directly regulates HIF-1β mRNA and protein levels, resulting in modulation of HIF-2α protein levels by preventing protein degradation [34,35]. Additional studies have also shown that SP1 (specificity protein 1)/3 and Egr-1 transcription factors and the STAT3 transcriptional activator can all regulate expression of HIF 1α RNA [36–41]. HIF-2α can be regulated by SP1/3 in 3T3-L1 mouse embryonic cells during adipocyte differentiation [42] and during cell progression by E2F1 in cancer [43]. Interestingly, HIF-2α has recently been shown to be sensitive to histone deacetylase (HDAC) inhibition in soft tissue sarcomas [44]. HDACs are components of several transcriptional repressor complexes, including the SIN3A complex [4].

Here, we show that SINHCAF links SIN3A function to hypoxia signaling. SINHCAF targets the HIF-2α promoter, resulting in histone deacetylation and gene silencing. SINHCAF is maintained at the HIF-2α promoter by the transcription factor SP1. Interestingly, SINHCAF is also required for optimal retention of SP1 at the HIF-2α promoter. Finally, SINHCAF and HIF-2α functions are inversely correlated in an *in vitro* angiogenesis assay and in the control of cellular viability/proliferation.

## Materials and methods

### Cell culture and treatments

All cell lines were maintained for no more than 30 passages and grown in Dulbecco's modified Eagle's medium or Roswell Park Memorial Institute medium containing 1% penicillin and streptomycin, supplemented with 10% fetal bovine serum (Sigma). Hypoxic treatments were 1% O_2_ unless otherwise stated and delivered using a Baker-Ruskinn InVivo2 300 hypoxia chamber. MG132 (Merck/Millipore) was dissolved in DMSO added for 6 h at final concentration of 10 µM. TSA (Trichostatin A; NEB, U.K.) was added to cells where indicated for 6 h at final concentration of 400 nM. Serum response experiments were performed as described in ref. [43]. Briefly, cells were transfected as described below, 24 h later, media were changed to low serum (0.5%) for an additional 24 h. Where indicated, full media (10%) were added for an additional 6 h prior to lysis.

### Small interfering RNA and plasmid transfection

Small interfering RNA (siRNA) transfections were performed using Interferin (Peqlab), and DNA transfections using TurboFect (Thermo). All reagents were used according to the manufacturer's instructions. SINHCAF expression constructs were described in ref. [1]. HIF-2α promoter fused to renilla luciferase construct was obtained from GeneCopoeia.

### siRNA sequences

Control, CAG UCG CGU UUG CGA CUG G [45]; HIF-2α, CAG CAU CUU UGA CAG U [45]; SINHCAF_1, CAG UAA ACU GCA GAA GGA A [1]; SINHCAF_2, GUC AGA UGA CGG CUC AGA U [1]; PHD2, GACGAAAGCCAUGGUUGCUUG [46]; E2F1, CGC UAU GAG ACC UCA CUG [47]; NFKB2, CAG CCU AAG CAG AGA GGC U [48]; SP1, CCU GGA GUG AUG CCU AAU A [49]; SP3, AGA CGA AGC UGG UAA UCU A; SIN3A, GGU CUA AGA GCU UAC UCA A [1]; HDAC1, GUU AGG UUG CUU CAA UCU A [1].

### Integrative analysis using public datasets

Analysis of A549 microarray [2] was performed using the GEO2R tool on the GEO website. The following ChIP (chromatin immunoprecipitation) sequencing datasets from the encode project [50,51] were downloaded from the NCBI GEO database, HeLa S3 RNA Pol II (GSM935395), A549 SIN3A (GSM1010882), and HeLa S3 H3K4me3 (GSM733682). Coverage tracks were generated using the Gvis R Bioconductor package [52].

### Immunoblots

Cells were lysed in RIPA buffer, 50 mM Tris–HCl (pH 8), 150 mM NaCl, 1% (v/v) NP40, 0.5% (v/v) Na-deoxycholate, 0.1% (v/v) SDS, and 1 tablet/10 ml *Complete*, Mini, EDTA-free protease inhibitors (Roche). SDS–PAGE and immunoblots were carried out using standard protocols.

Antibodies were used as follows: SINHCAF [1], HIF-2α (PA1-16510, Thermo Scientific; 7096, Cell Signaling), β-actin (3700, Cell Signaling), HIF-1α (610958, BD Biosciences), HIF-1β (3718, Cell Signaling), PHD2 (Bethyl A300-322A; 4835, Cell Signaling); p52 (05-361, Merck/Millipore), E2F1 (3742, Cell Signaling), SP1 (07-645, Merck/Millipore), SP3 (sc-644, Santa Cruz Biotech), Sin3A (sc-994, Santa Cruz Biotech; 8056, Cell Signaling); HDAC1 (17-10199; Merck/Millipore), AcH3 (acetylated histone H3; 06-599, Merck/Millipore), p62/STQM (610832, BD Biosciences), poly-ADP ribose polymerase (PARP) (9532, Cell Signaling), cleaved PARP (9541, Cell Signaling), caspase-3 (9662, Cell Signaling), cleaved caspase-3 (9661, Cell Signaling), and LC3B (3868, Cell Signaling).

### Immunoprecipitation

For immunoprecipitation of endogenous SINHCAF, cells lysed in lysis buffer [10 mM Tris–HCl (pH 7.5), 150 mM NaCl, 1% (v/v) Triton X-100, 20% (v/v) glycerol, and 1 tablet/10 ml *Complete*, Mini, EDTA-free protease inhibitors (Roche)].

Cleared cell lysate was rotated overnight at 4°C with 2 µg of anti-SINHCAF antibody and then for an additional 1 h 30 min after adding protein G-Sepharose (Generon). Immobilized antigen–antibody complex was then washed three times with PBS and eluted in 20 µl of Laemli (2×SDS buffer) buffer.

### Quantitative reverse transcription–polymerase chain reaction

RNA was extracted using the peqGOLD total RNA kit (Peqlab), according to the manufacturer's instructions, and reverse transcribed using the QuantiTect Reverse Transcription Kit (Qiagen). For quantitative PCR, Brilliant II Sybr green kit (Statagene/Agilent), including specific MX3005P 96-well semi-skirted plates, was used to analyze samples on the MX3005P qPCR platform (Stratagene/Agilent). Actin was used as a normalizing agent in all experiments. The following primers were used for RT–PCR:

**Actin:** F: 5′-CTGGGAGTGGGTGGAGGC-3′
**Actin:** R: 5′-TCAACTGGTCTCAAGTCAGTG-3′

**HIF-2α:** F: 5′-TTTGATGTGGAAACGGATGA-3′
**HIF-2α:** R: 5′-GGAACCTGCTCTTGCTGTTC-3′

**HIF-1α:** F: 5′-CAT AAA GTC TGC AAC ATG GAA GGT-3′
**HIF-1α:** R: 5′-ATT TGA TGG GTG AGG AAT GGG TT-3′

**SINHCAF:** F: 5′-CCTGTGCCTCCCTTCATATT-3′
**SINHCAF:** R: 5′-CAGGGTCTTGCCTATCCTAAAG-3′

**SP1:** F: 5′-ACC AGG CTG AGC TCC ATG AT-3′
**SP1:** R: 5′-CCT CAG TGC ATT GGG TAC TTC-3′

### ChIP-PCR assays

ChIP assays were performed as described previously [48]. Briefly, proteins and chromatin were cross-linked with 1% formaldehyde at room temperature for 10 min. Glycine was added to a final concentration of 0.125 M for 5 min to quench the reaction. Cells were harvested into 400 µl of lysis buffer [1% SDS, 10 mM EDTA, 50 mM Tris–HCl (pH 8.1), 1 mM PMSF, 1 µg/ml leupeptin, and 1 µg/ml aprotinin] and left on ice for 10 min. Samples were then sonicated at 4°C eight times for 15 s with a 30 s gap between each sonication at 50% amplitude (Sonics Vibra-Cell # VCX130). Supernatants were recovered by centrifugation (12 000 rpm for 10 min at 4°C) before 10% of each sample was stored as input. Remaining samples were split into 120 µl aliquots before being diluted 10-fold in dilution buffer [1% Triton X-100, 2 mM EDTA, 150 mM NaCl, and 20 mM Tris–HCl (pH 8.1)]. Diluted samples were pre-cleared for 2 h at 4°C by incubating with 2 µg of sheared salmon sperm DNA and 20 µl of protein G-Sepharose (50% slurry).

Immunoprecipitations were performed overnight on the remaining sample with 2 µg of antibody, with the addition of Brij 35 detergent to a final concentration of 0.1%. Immune complexes were captured by incubation with 40 µl of protein G-Sepharose (50% slurry) and 2 µg of salmon sperm DNA for 1 h at 4°C. The immunoprecipitates were washed sequentially for 5 min each at 4°C in wash buffer 1 [0.1% SDS, 1% Triton X-100, 2 mM EDTA, 20 mM Tris–HCl (pH 8.1), and 150 mM NaCl], wash buffer 2 [0.1% SDS, 1% Triton X-100, 2 mM EDTA, 20 mM Tris–HCl (pH 8.1), and 500 mM NaCl], and wash buffer 3 [0.25 M LiCl, 1% Non-idet P-40, 1% deoxycholate, 1 mM EDTA, and 10 mM Tris–HCl (pH 8.1)]. Beads were washed twice with Tris–EDTA buffer and eluted with 120 µl of elution buffer (1% SDS and 0.1 M NaHCO_3_). Cross-links were reversed by incubation with 0.2 M NaCl at 65°C overnight, and Proteinase K (20 µg each), 40 mM Tris–HCl (pH 6.5), and 10 mM EDTA for 1 h at 45°C were used to remove protein. DNA was purified using a PCR-product purification kit according to the manufacturer's instructions (NBS Biologicals #NBS363). Three microliters of DNA were used for qPCR with the following primers for the HIF-2α promoter.

F: 5′-TCCTCCCAGTCACCTTTCT-3′
R: 5′-TGGTGAAGGTCCGAGGT-3′

qPCR results are expressed relatively to input material, as mean and SEM of a minimum of three independent experiments.

### Tube formation assay

Tube formation assays were performed using HUVECs and µ-slides (i-Bidi) as described in ref. [53]. Briefly, 10 µl of Geltrex (Invitrogen) was added to the inner well and allowed to set at 37°C. A total of 6 × 10^5^ HUVECs were suspended in 300 µl media composed of one-third complete Medium200PRF and two-third conditioned media from assay condition. Fifty microliters of this mixture containing 1 × 10^5^ cells were plated in the outer well of the µ-slide. Cells were allowed organize on the recombinant basement membrane by incubation overnight at 37°C prior to images being acquired by microscopy. The number and length of tube branches were calculated using the images acquired in Image J software (NIH).

### Colony formation assay

HeLa and DLD-1 cells were transfected with control or SINHCAF, HIF-2α, or a combination of the two siRNAs for 24 h prior to trypsination. Cells were counted and plated on to six-well plates at the density of 5000 or 10 000 cells per well. Cells were allowed to grow for 6 days prior to staining with Crystal Violet. Plates were imaged and colonies counted using Image J.

### Proliferation assay

These assays were performed as described in ref. [54]. Briefly, HeLa cells were transfected with control or SINHCAF siRNAs for 24 h prior to trypsination. Cells were counted and plated on to six-well plates at the density of 50 000 cells per well. Cells were counted 24 and 48 h later using a hemocytometer in triplicate. Results are expressed as an average of total cells counts in each day and represent a three independent experiment.

### Cell cycle analysis

Cell cycle analysis was performed as described in ref. [43]. Briefly, cells were trypsinized, media and PBS washes collected in the same tube prior to fixation with 70% ethanol. Samples were then frozen at −20°C for a minimum of 1 day. Fixed cells were washed with PBS and stained using the Cell Cycle analysis Kit from Guava (Millipore 4500-0220) and analyzed in a Guava^®^ easycyte HT (Millipore) cytometer.

### Statistical analysis

Data analysis was performed using SigmaPlot v.12.0 (Systat Software, Inc., CA, U.S.A.). One-way analysis of variance (ANOVA) with the Holm–Sidak pairwise comparison was used to compare results between the groups, and data are presented as category mean ± standard error of the mean unless otherwise stated. All statistical tests were two-sided and statistical significance denoted as follows: *P ≤ 0.05, **P ≤ 0.01, ***P ≤ 0.001.

### Additional experimental procedures

Additional experimental procedures, such as luciferase assays, have been described previously [55].

## Results

### SINHCAF and SIN3A depletion result in increased HIF-2α levels

**Figure 1.**
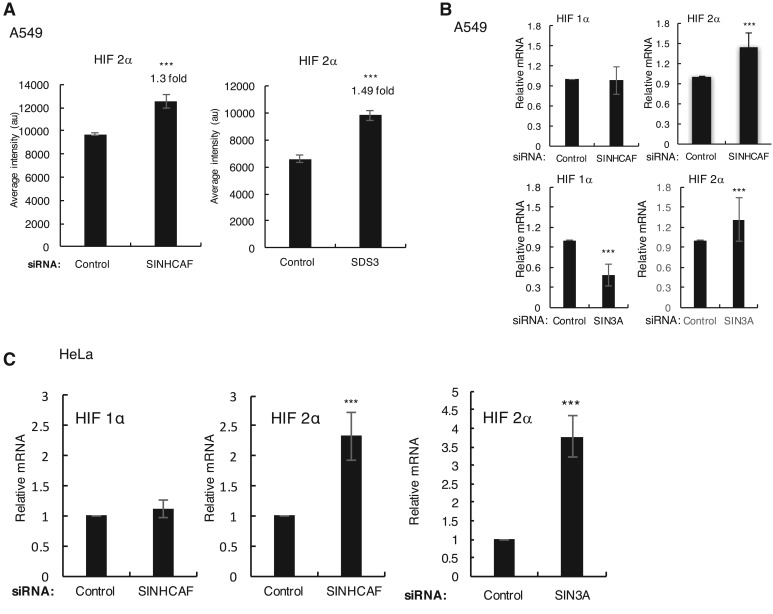
SINHCAF is a repressor of HIF-2α mRNA. (**A**) Microarray analysis levels for HIF-2α following SINHCAF and SDS3 in A549 cells. (**B**) qPCR validation using A549 cells, and independent sets of siRNA oligonucleotides for SINHCAF and SIN3A. Graphs depict mean + SEM. *P ≤ 0.05, **P ≤ 0.01, ***P ≤ 0.001. (**C**) qPCR validation using HeLa cells, and siRNA oligonucleotides for SINHCAF and SIN3A. Graphs depict mean + SEM. *P ≤ 0.05, **P ≤ 0.01, ***P ≤ 0.001.

SINHCAF has been identified as a component of the SIN3A/HDAC complex [1,2] and to contribute to transcriptional control of Cyclin D1 [1]. Microarray analysis of SINHCAF and SIN3A regulated genes in A549 cells has identified co-regulated genes within the TGF-β signaling pathway [2]. We have further analyzed this dataset and identified HIF-2α (EPAS1), as one of the genes shared by both SINHCAF and SIN3A (Figure 1A). We confirmed this observation by qPCR, using independent siRNAs for SINHCAF and SIN3A (Figure 1B). Interestingly, this regulation was specific for HIF-2α, as levels of HIF-1α were unaltered by SINHCAF depletion or reduced by SIN3A knockdown in A549 cells (Figure 1B). Similar results were also observed when we analyzed HeLa cells (Figure 1C and Supplementary Figure S1A).

**Figure 2.**
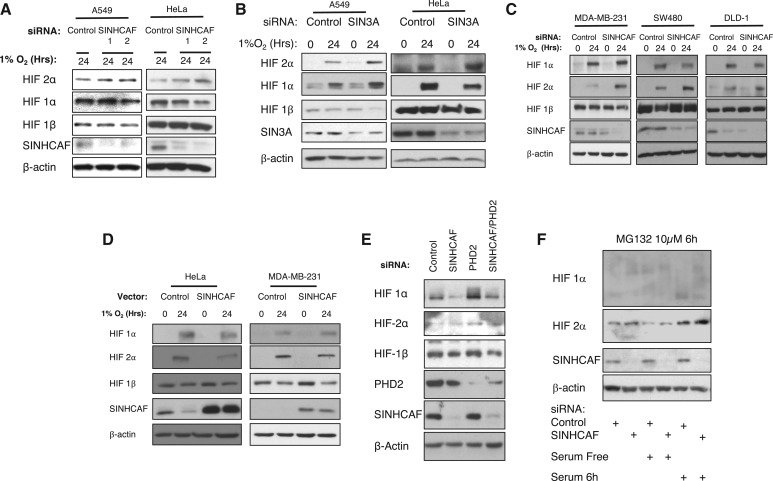
SINHCAF is a repressor of HIF-2α protein in multiple cell lines. (**A**) Control or one of the two SINHCAF [1/2] siRNA oligonucleotides were transfected into A549 and HeLa cells cultured in the presence of hypoxia for 24 h. Lysed samples were analyzed by immunoblot for expression of HIF system isoforms and SINHCAF. (**B**) Control or SIN3A siRNA oligonucleotides were transfected to A549 and HeLa cells cultured in normoxia or hypoxia for 24 h. Lysed samples were analyzed by immunoblot for expression of HIF system isoforms and SIN3A. (**C**) Expression of HIF-2α following knockdown of SINHCAF and exposure to hypoxia for 24 h was determined in breast MDA-MB-231 and two colorectal (SW480, DLD-1) cancer cell lines. (**D**) SINHCAF was overexpressed in HeLa and MDA-MB-231 cells with or without exposure to hypoxia for 24 h. Lysed samples were analyzed by immunoblot for expression of HIF system isoforms and SINHCAF. (**E**) Control, SINHCAF, and PHD2 were singly or doubly knocked down in HeLa cells and expression of the HIF system isoforms was determined by immunoblot. (**F**) Control and SINHCAF siRNA oligonucleotides were transfected into HeLa cells. Where indicated, cells were starved or serum for 24 h, or serum-starved and serum-added for the final 6 h prior to harvest. MG132 was added for the final 6 h in all conditions. Representative images from at least three experiments are shown.

To determine if SINHCAF is a novel regulator of HIF-2α protein expression, A549 and HeLa cells were transfected with control or two SINHCAF siRNA oligonucleotides in order to examine the effect of SINHCAF knockdown on the expression of the HIF isoforms (Figure 2A and Supplementary Figure S1B). Knockdown of SINHCAF resulted in an increase in HIF-2α, but not in HIF-1α or HIF-1β protein expression following exposure to hypoxia for 24 h. Similar results were also observed when SIN3A was depleted by siRNA, with elevated levels of HIF-2α occurring in both HeLa and A549 cells (Figure 2B and Supplementary Figure S1C). Given the importance of maintaining the levels of HIF transcription factors, small changes at mRNA and protein levels can result in significant biological effects. This has been demonstrated by our previous work, and that of others, using cancer cells but also the model organism *Drosophila melanogaster* [11,20,30,33,43,53].

To determine the penetrance of this effect, similar experiments were performed in multiple cell lines. The loss of SINHCAF resulted in significant increases in HIF-2α with little or no change to HIF-1α protein following exposure to hypoxia in breast cancer cells (MDA-MB-231) and two colorectal (SW480, DLD-1) cell lines (Figure 2C). In addition, overexpression of control or SINHCAF cDNA plasmids in cells was performed to determine if gain-of-function experiments would lead to the opposite effect on HIF-2α levels. Overexpression of SINHCAF resulted in a significant decrease in HIF-2α protein following exposure to hypoxia for 24 h in both HeLa and MDA-MB-231 cells, confirming that the siRNA results are not a technical artifact but also the responsiveness of the system (Figure 2D).

Given that our protein analysis has been performed under hypoxic conditions, we next investigated if SINHCAF could control HIF-2α protein levels following different stimuli. To this end, we performed depletion of PHD2, the main regulator of HIF-α isoforms, in the presence or absence of SINHCAF. Both SINHCAF and PHD2 depletion resulted in modest increases in normoxic levels of HIF-2α (Figure 2E and Supplementary Figure S1D). However, when combined, there was no additive or synergistic effect, a puzzling result, which we cannot fully explain. On the other hand, HIF-1α levels were up-regulated in the absence of PHD2, but once again, this effect was lost when both SINHCAF and PHD2 were depleted (Figure 2E).

To further investigate the effects of SINHCAF in the control of normoxic HIF-2α, we next investigated how SINHCAF alters the levels of HIF-2α in response to serum starvation/addition (Figure 2F). We have previously shown that HIF-2α mRNA levels do increase in response to serum addition [43]. To be able to fully visualize normoxic HIF-2α, we also included the addition of MG132 to cells, prior to lysis. This analysis revealed that SINHCAF depletion does result in increased levels of HIF-2α protein, in normoxic conditions but also in response to serum addition. Furthermore, the increase in HIF-2α protein in the absence of SINHCAF is blunted in the absence of serum (Figure 2F). Taken together, our results are consistent with SINHCAF control of HIF-2α mRNA being observed also at the protein level of this important transcription factor.

### SINHCAF regulates the HIF-2α promoter directly and is important for HDAC1 recruitment

**Figure 3.**
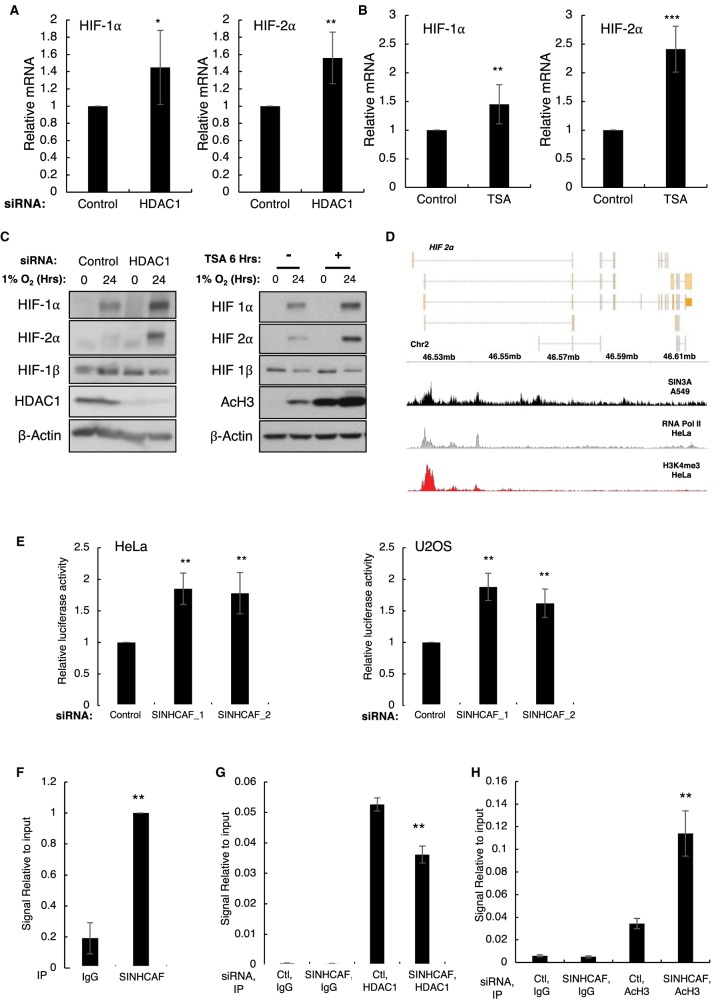
SINHCAF, but not HDAC1, is a specific repressor of HIF-2α promoter. (**A**) HDAC1 or non-targeting siRNA oligonucleotides were transfected to HeLa cells prior to RNA extraction. RNA expression of the HIF-α isoforms was determined by qPCR. Graphs depict mean + SEM. *P ≤ 0.05, **P ≤ 0.01, ***P ≤ 0.001. (**B**) HeLa cells were treated with TSA for 6 h and RNA was extracted. qPCR analysis for the levels of HIF-1α and HIF-2α was performed. Graphs depict mean + SEM. *P ≤ 0.05, **P ≤ 0.01, ***P ≤ 0.001. (**C**) HDAC1 or non-targeting siRNA oligonucleotides were transfected to HeLa cells cultured in the presence or absence of hypoxia for 24 h (right panel). HeLa cells were treated with TSA for 6 h and exposed or not to 24 h of 1% O_2_, prior to lysis (left panel). Lysed samples were analyzed by immunoblot for expression of HIF system isoforms. (**D**) Coverage track analysis of SIN3A ChIP-sequencing in A549 cells at the HIF-2α gene. RNA Pol II and H3K4me3 coverage tracks for HeLa are also shown. (**E**) HeLa and U2OS cells stably expressing an HIF-2α promoter–renilla luciferase reporter construct were transfected with control or one of two SINHCAF [1/2] siRNA oligonucleotides and luciferase activity was measured. Graphs depict mean + SEM. *P ≤ 0.05, **P ≤ 0.01, ***P ≤ 0.001. (**F**) ChIP for SINHCAF was performed in HeLa cells and HIF-2α promoter occupancy was analyzed by qPCR. Graphs depict mean + SEM. *P ≤ 0.05, **P ≤ 0.01, ***P ≤ 0.001. (**G**) ChIP for HDAC1 was performed in HeLa cells that had been transfected with control or SINHCAF siRNA oligonucleotides. An assessment of HIF-2α promoter occupancy was performed by qPCR. Graphs depict mean + SEM. *P ≤ 0.05, **P ≤ 0.01, ***P ≤ 0.001. (**H**) Change in histone H3 acetylation at the HIF-2α promoter was analyzed following SINHCAF knockdown by qPCR. Graphs depict mean + SEM. *P ≤ 0.05, **P ≤ 0.01, ***P ≤ 0.001. See also Supplementary Figure S1.

Very little is known about the regulation of the HIF-2α gene, apart that it can be regulated by E2F1 [43], its promoter can be methylated [56,57], and that HDACs can repress both HIF-2α and HIF-1α genes [44]. We could also confirm that indeed depleting HDAC1 or treating cells with the class I and II HDAC inhibitor TSA resulted in increased levels of HIF-1α and HIF-2α, observed at mRNA (Figure 3A and B) and protein level (Figure 3C).

Analysis of published datasets revealed that the HIF-2α promoter was identified in an SIN3A ChIP-sequencing experiment (Figure 3D). To determine if the HIF-2α promoter was being specifically regulated by SINHCAF, we analyzed HIF-2α promoter constructs in the presence or absence of SINHCAF. HeLa and U2OS cells, stably expressing a renilla luciferase-HIF-2α promoter construct, were transfected with control or one of two SINHCAF siRNA oligonucleotides (Figure 3E). In accordance with our previous results investigating HIF-2α mRNA levels, SINHCAF knockdown resulted in a significant increase in renilla luciferase activity, suggesting that the HIF-2α promoter is regulated by SINHCAF. A decrease in promoter activity was also observed when SINHCAF was overexpressed (Supplementary Figure S2A).

To test if SINHCAF can control the HIF-2α promoter directly, ChIP for SINHCAF and qPCR analysis using primers directed toward the HIF-2α promoter was performed. This analysis revealed a significant enrichment of SINHCAF present at the HIF-2α promoter (Figure 3F). We had previously demonstrated that SINHCAF was required for HDAC1 recruitment to the Cyclin D1 promoter [1]. We thus determined if a similar mechanism was in place for the HIF-2α promoter. We analyzed the levels of HDAC1 and AcH3 in the presence or absence of SINHCAF (Figure 3G,H) present at the HIF-2α promoter. This analysis revealed that SINHCAF depletion resulted in a decrease in the levels of HDAC1 detected at the HIF-2α promoter (Figure 3G) with a correspondent increase in histone acetylation (Figure 3H), consistent with the proposed mechanism of SIN3A/HDAC1 recruitment to the HIF-2α promoter. Taken together, these results demonstrate that SINHCAF is important for HDAC1 recruitment to the HIF-2α promoter, controlling transcription of the HIF-2α gene.

### SINHCAF represses HIF-2α promoter via SP1

**Figure 4.**
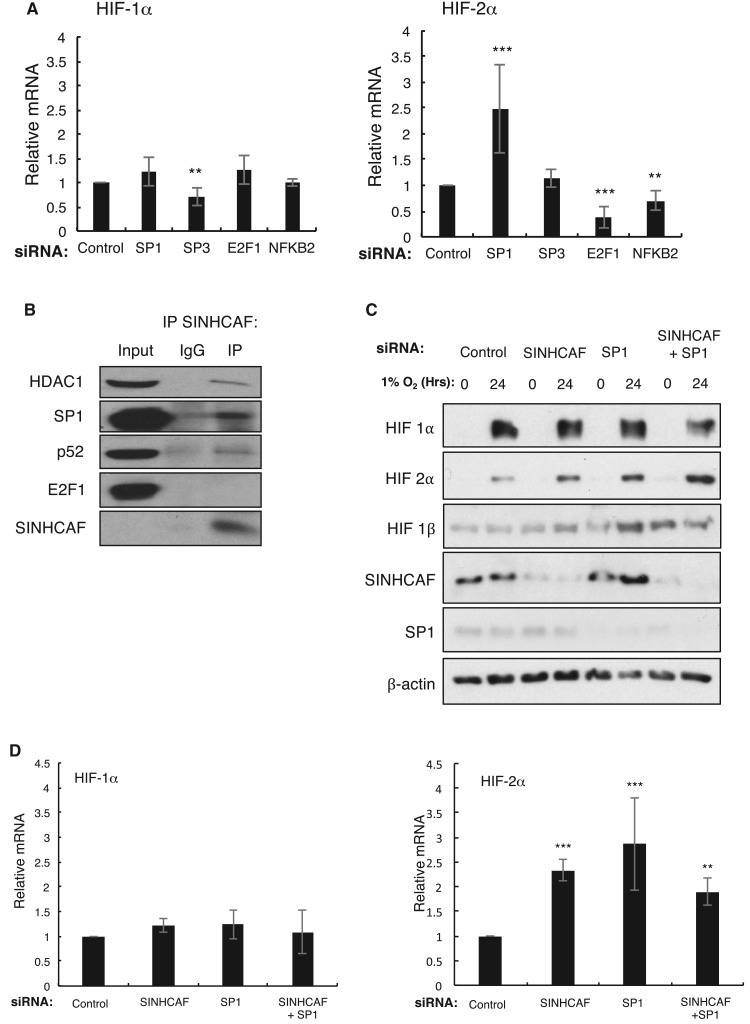
SINHCAF regulates HIF-2α expression in cooperation with the sequence-specific transcription factor SP1. (**A**) siRNA knockdown of multiple transcription factors was performed in HeLa cells to examine its effect on basal expression of HIF-α. Graphs depict mean + SEM. *P ≤ 0.05, **P ≤ 0.01, ***P ≤ 0.001. (**B**) Endogenous SINHCAF immunoprecipitations in HeLa cells were conducted to determine SINHCAF interaction with SP1, p52, and E2F1. (**C**) Control, SINHCAF, and SP1 were singly or doubly knocked down in HeLa cells with or without treatment with hypoxia for 24 h and expression of the HIF system isoforms was determined by immunoblot. (**D**) RNA expression of the HIF-α isoforms was examined by qPCR following single or multiple knockdown of control, SINHCAF, and SP1. Representative images from at least three experiments are shown. *N* = 3. Graphs depict mean + SEM. *P ≤ 0.05, **P ≤ 0.01, ***P ≤ 0.001. See also Supplementary Figure S2.

The results obtained with HDAC inhibition suggest that SINHCAF provide the specificity of targeting a specific HIF-α isoform. Bioinformatic analysis of the HIF-2α promoter revealed potential binding sites for NF-κB, E2F1, and SP1 [43]. To determine the possible contribution of the predicted transcription factors to the control of HIF-2α expression, each factor was sequentially knocked down by siRNA in HeLa cells. We also included SP3, given its close overlap with SP1. The loss of SP3 was shown to reduce the basal expression of HIF-1α RNA with SP3 acting as an activator of basal HIF-1α RNA expression (Figure 4A and Supplementary Figure S3A). SP1, E2F1, and p52 were all shown to affect the basal expression of HIF-2α mRNA. Here, E2F1 and p52 function as activators, while SP1 functions as a repressor of basal HIF-2α mRNA expression (Figure 4A).

We next determined if any of these transcription factors was able to interact with SINHCAF. To this end, immunoprecipitation reactions were performed for SINHCAF and levels of the putative HIF-2α transcriptional regulators were investigated. SP1 and p52 were found to interact with SINHCAF in HeLa cells (Figure 4B). This result in combination with our previous published studies [1,48,58] suggests that SINHCAF might interact with p52 to control Cyclin D1 promoter. Based on the observation that only SP1 was able to repress the levels of HIF-2α mRNA, and was found to interact with SINHCAF in co-immunoprecipitation experiments, we hypothesized that SP1 is the transcription factor responsible for the recruitment of SINHCAF and the SIN3A–HDAC complex to the HIF-2α promoter. To test this hypothesis, control, SINHCAF, and SP1 were singly or doubly knocked down by siRNA in HeLa cells, with or without exposure to hypoxia for 24 h. Consistent with the mRNA analysis, none of the depletions resulted in a significant change in HIF-1α protein expression (Figure 4C). However, loss of SINHCAF or SP1 resulted in a significant increase in HIF-2α protein expression compared with control (Figure 4C). Interestingly, double depletion of SINHCAF and SP1 had similar effects to single knockdown approaches, suggesting that both proteins work in the same pathway. This was also confirmed by mRNA expression analysis through qPCR in HeLa cells. Single or combined loss of SINHCAF and SP1 resulted in a significant but non-additive increase in HIF-2α but not in HIF-1α mRNA expression (Figure 4D). These results suggest that both SINHCAF and SP1 act in the same pathway to repress HIF-2α.

### Complex occupancy at the HIF-2α promoter

**Figure 5.**
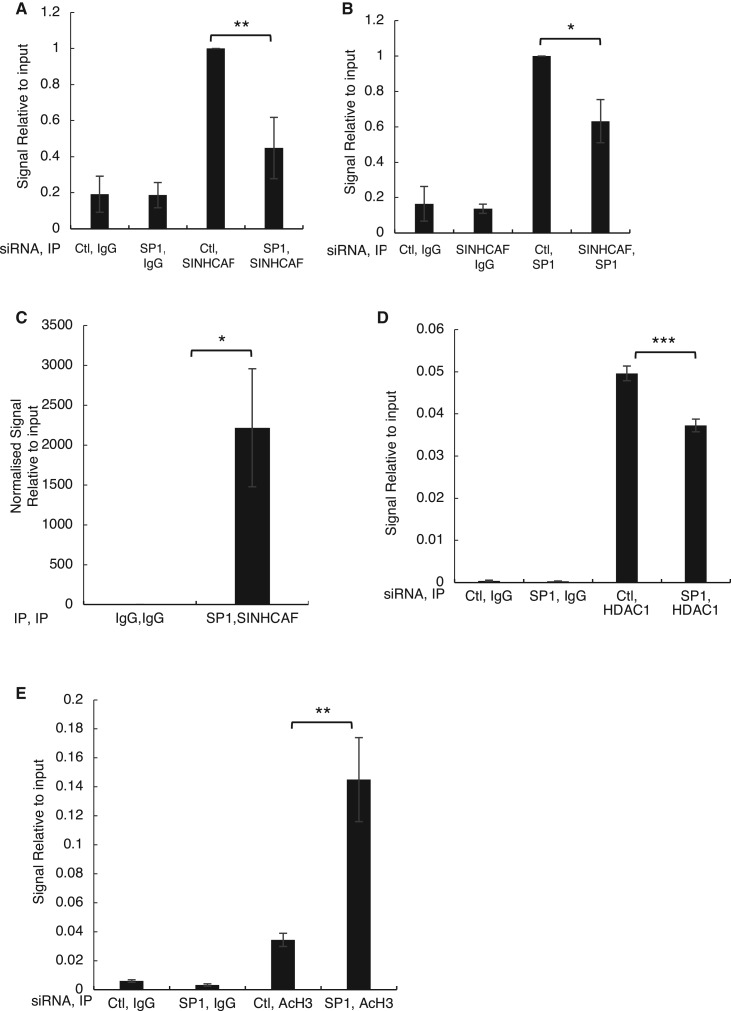
SP1 is required for SINHCAF occupancy at the HIF-2α promoter. (**A**) ChIP for SINHCAF was performed in HeLa cells that had been transfected with control, or SP1 siRNA oligonucleotides. Signal relative to input for control samples was set to 1. Additional experimental conditions were compared with control, as shown. Graphs depict mean + SEM. *P ≤ 0.05, **P ≤ 0.01, ***P ≤ 0.001. (**B**) ChIP for SP1 was performed in HeLa cells that had been transfected with control, or SINHCAF siRNA oligonucleotides. Signal relative to input for control samples was set to 1. Additional experimental conditions were compared with control, as shown. Graphs depict mean + SEM. *P ≤ 0.05, **P ≤ 0.01, ***P ≤ 0.001. (**C**) Re-ChIP SP1/SINHCAF confirmed co-occupancy at the HIF-2α promoter. Graphs depict mean + SEM. *P ≤ 0.05, **P ≤ 0.01, ***P ≤ 0.001. (**D**) HDAC1 promoter occupancy in the presence or absence of SP1. Graphs depict mean + SEM. *P ≤ 0.05, **P ≤ 0.01, ***P ≤ 0.001. (**E**) Change in histone H3 acetylation at the HIF-2α promoter was quantified following SP1 knockdown. *N* = 3. Graphs depict mean + SEM. *P ≤ 0.05, **P ≤ 0.01, ***P ≤ 0.001.

To test if SP1 can control the recruitment of SINHCAF to the HIF-2α promoter, ChIP was performed in HeLa cells, proficient or depleted of SP1. siRNA depletion of SP1 resulted in a significant reduction in SINHCAF promoter occupancy (Figure 5A). Interestingly, SP1 occupancy was similarly affected by SINHCAF knockdown (Figure 5B), suggesting an important interaction between these two factors. To determine if both proteins occupy the same site, ChIP/re-ChIP for SP1/SINHCAF was performed. This analysis demonstrated that both proteins do co-occupy the HIF-2α promoter (Figure 5C). We next investigated if SP1 is also important for the recruitment of HDAC1 to the HIF-2α promoter and the functional consequences of this. Levels of HDAC1 and AcH3 in the presence or absence of SP1 were analyzed (Figure 5D,E). This analysis revealed that SP1 depletion resulted in locally increased histone acetylation consistent with the decrease in HDAC1 recruitment to the HIF-2α promoter (Figure 5D,E). Taken together, these results demonstrate that SP1 is important for SINHCAF/HDAC1 recruitment to the HIF-2α promoter.

### SINHCAF controls HIF-2α activity in cells

**Figure 6.**
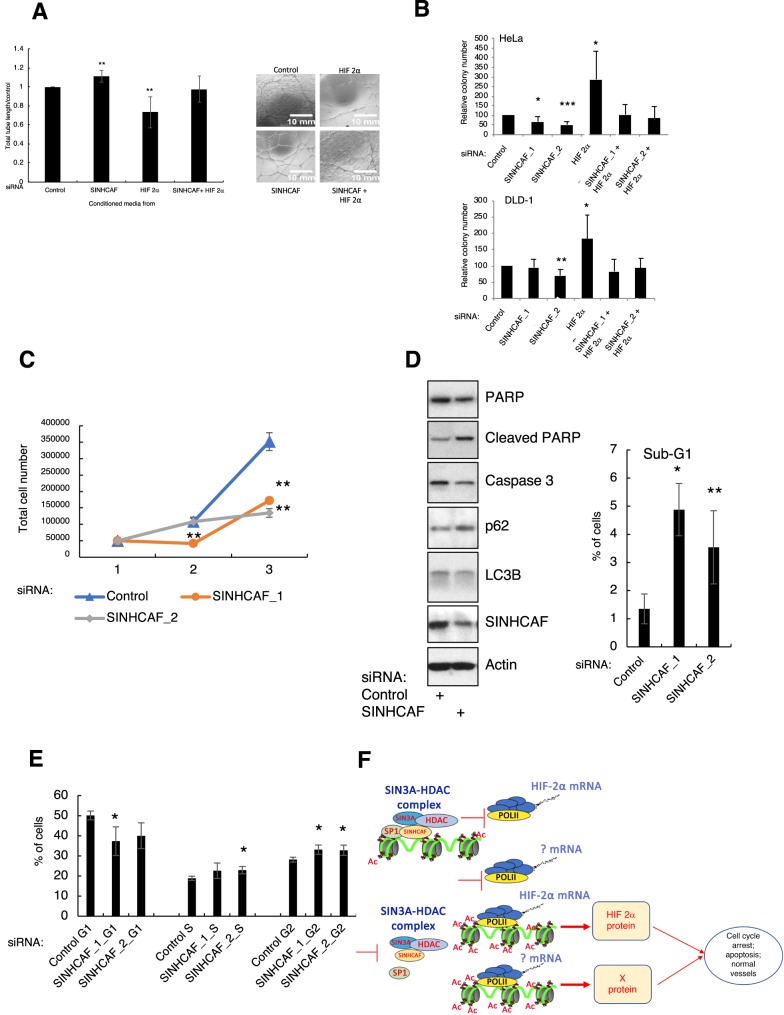
Functional significance of SINHCAF-mediated HIF-2α repression. (**A**) Tube formation: conditioned media were collected from DLD-1 cells treated with single or double knockdown of control, SINHCAF, or HIF-2α, and cultured in hypoxia for 24 h. HUVECs were cultured in the presence of recombinant basement membrane and conditioned media for 24 h. Total tube length was measured by Image J macros, and representative images are shown. *N* = 3. Graphs depict mean + SEM. *P ≤ 0.05, **P ≤ 0.01, ***P ≤ 0.001. (**B**) Colony formation: HeLa and DLD-1 cells were seeded for colony formation following siRNA transfection. Relative colony number at day 7 for each cell line is shown. *N* = 3. Graphs depict mean + SEM. *P ≤ 0.05, **P ≤ 0.01, ***P ≤ 0.001. (**C**) Control or one of two SINHCAF [1/2] siRNA oligonucleotides were transfected into HeLa cells for 24 h prior to trypsinization and equal number of cells plated on day 1. Cells were counted for two subsequent days. Graph depicts total cell number as the average and SD of three independent experiments. **P ≤ 0.01. (**D**) Control or SINHCAF siRNA oligonucleotides were transfected to HeLa cells for 48 h. Lysed samples were analyzed by immunoblot for expression of apoptotic and autophagy markers. Graphic depicts the percentage of sub-G1 cells present when analyzed by flow cytometry and represents the average and SEM. *P ≤ 0.05. (**E**) Control or one of two SINHCAF [1/2] siRNA oligonucleotides were transfected into HeLa cells for 48 h prior to cell fixation. Cells were analyzed by flow cytometry for the cell cycle distribution. Graph depicts the percentage of cells present in each stage of the cell cycle, and it is the average and SD of three independent experiments. *P ≤ 0.05, **P ≤ 0.01. (**F**) Proposed model for SINHCAF function over HIF-2α and its biological functions.

HIF transcription factors have important functions in tumor progression, such as controlling proliferation and angiogenesis [59–63]. To determine the importance of SINHCAF-mediated control of HIF-2α functions in cancer, *in vitro* cellular assays of angiogenesis were performed. Human umbilical vein endothelial cell (HUVEC) tube formation is analogous to angiogenesis *in vivo*. Conditioned media from DLD-1 tumor cells that had been treated with single or double siRNA knockdown of control, SINHCAF, and HIF-2α and treated with hypoxia for 24 h were collected. HUVECs were encouraged to form tubes in the presence of conditioned media for 24 h. The loss of SINHCAF resulted in a significant increase in total tube length following treatment with conditioned media, loss of HIF-2α resulted in a significant reduction in the same parameter, and combined depletion recovered the effects observed with individual SINHCAF and HIF-2α depletions (Figure 6A).

An important cellular function deregulated in cancer is proliferation. To determine how SINHCAF control over HIF-2α alters cell proliferation, HeLa and DLD-1 cells were transfected with control, SINHCAF, HIF-2α, or SINHCAF and HIF-2α siRNAs, and cell proliferation was analyzed using colony-forming assays. This analysis revealed that SINHCAF depletion resulted in a decreased number of colonies, while HIF-2α depletion significantly increased colony formation in both cell types (Figure 6B). Importantly, the effect of SINHCAF depletion on colony numbers was shown to be dependent on HIF-2α (Figure 6B and Supplementary Figure S4A), demonstrating the importance of SINHCAF control over HIF-2α levels and function.

To investigate the functional significance of SINHCAF depletion in additional biological responses, we performed a basic proliferation assay by counting the total cell number through time when cells possessed or not SINHCAF protein (Figure 6C). This analysis revealed that cell proliferation was significantly reduced 3 days following transfection of cells with SINHCAF siRNA oligonucleotides. To further determine the cause of these defects in proliferation and colony formation, we investigated levels of apoptosis (Figure 6D) and cell cycle distribution (Figure 6E). HeLa cells depleted of SINHCAF for 48 h revealed increased levels of apoptosis markers such as reduction caspase 3 levels, reduction of total PARP levels, and increased levels of cleaved PARP (Figure 6D). This was also observed in the percentage of cells present in the sub-G1 population detected in the flow cytometry analysis (Figure 6D, graphic). Markers for autophagy were slightly reduced, with decreased levels of LC3B, and increased levels of p62. These data suggest that reduction of SINHCAF results in reduction of cellular viability. Finally, cell cycle analysis revealed that SINHCAF depletion resulted in a modest but significant increase in cell arrested in the G2/M stage of the cell cycle (Figure 6E). Taken together, these results suggest that SINHCAF can control both cell cycle and cell death pathways.

## Discussion

In the present study, we show that SINHCAF, a relatively unknown protein, controls the expression of the HIF-2α gene. We found that SINHCAF acts to epigenetically silence expression of the HIF-2α gene through recruitment of the SIN3–HDAC co-repressor factor. This complex regulates HIF-2α expression via its interaction with the transcription factor SP1. SINHCAF expression is linked to functional cellular changes in angiogenesis (tube formation), viability, proliferation, and cell cycle *in vitro* (Figure 6F).

Evidence is emerging to suggest that control of the basal expression of the HIF system is as important as its hypoxia-responsive post-translational degradation [64]. Changes in basal expression of the HIF-α isoforms are important in determining tissue-specific, hypoxia-inducible gene expression and are also important in the progression of multiple types of disease including cancer [30,34,35,64–68]. Basal HIF expression appears to be regulated by a transcriptional rheostat that is designed to allow an organism to respond in a heterogeneous manner to changes in oxygen supply and demand through space and time. The HIF system is set differently in different cells, in a manner that is appropriate for the physiological control of oxygen homeostasis [69]. Our own laboratory has demonstrated the importance of regulation of the expression of HIF-1α previously. We demonstrated that the ATP-dependent chromatin remodeling complex SWI/SNF directly affects both the expression of HIF 1α and its ability to transactivate target genes [68].

The specific nature of the mechanism involving SINHCAF would allow selective repression of HIF-2α. This proposed mechanism of transcriptional regulation by chromatin modification is one established mechanism for the heritable control of transcription that would be capable of specifically controlling the temporal and spatial transcription of HIF-2α, a phenomenon already seen in several cell types. SINHCAF repression was shown to be specific for HIF-2α, while HDAC1 repression is seen for both HIF-2α and HIF 1α. SINHCAF thus acts as a specificity factor for HDAC-mediated control of the HIF genes.

HIF-2α repression has been shown to be important for progression of the disease [70]. *In vitro* analysis of SINHCAF and HIF-2α regulated cellular responses demonstrated their co-dependency. Our results demonstrate that indeed, SINHCAF depletion prevents colony formation, while HIF-2α depletion induced a significant increase in colonies formed. Again, these responses were reversed upon co-depletion. Furthermore, SINHCAF was seen to repress tube formation via HIF-2α with total recovery achieved following co-depletion. This implies that SINHCAF depletion results in an increased quality of blood vessels, an important aspect required for tumor suppression [71]. In addition, we observed that cells depleted of SINHCAF arrest in the G2/M stage of the cell cycle. This is an intriguing observation, given that HIF-2α is known to co-operate with c-Myc to drive the cell cycle in hypoxic cells [72]. However, our results are set in a normoxic setting and suggest that cells are accumulating in G2/M possibly due to S-phase defects. This is consistent with our previous results where we could determine that cells depleted from SINHCAF progressed faster through S-phase [1]. However, a more detailed analysis of the SINHCAF-depleted cell, regarding replicative stress, would be required to fully investigate this possibility.

HIF-2α repression can drive tumor progression indirectly through loss of expression of HIF-2α-dependent tumor suppressors. In support of these proposals, HIF-2α, but not HIF-1α deletion, has been shown to increase tumor growth and progression secondary to loss of the tumor suppressor secretoglobin 3a1 protein in a KRAS-driven lung tumor model [73,74]. In addition, HIF-2α was proposed to act as a tumor suppressor in a glioma rat model [75]. However, HIF-2α functions are very cell type-specific, since it is clear it acts as a tumor promoter in renal cancer [76,77]. Furthermore, similar tissue specificity has been observed for HIF-1α, which acts as tumor suppressor in renal cancer [78], but as a tumor promoter in breast cancer [79]. Our findings with SINHCAF provide another possible mechanism by which HIF-1α and HIF-2α are differentially regulated, strongly suggesting that indeed, non-canonical mechanisms control the HIF system, confer tissue/cell specificity, and influence the mode of action for these transcription factors.

The mechanism for the epigenetic regulation of HIF-2α by SINHCAF described in this manuscript represents an exciting and potentially druggable future therapeutic target. In the physiological environment, these mechanisms may play a more important role in the tissue-specific expression and kinetic response of the HIF-α isoforms than is currently understood. Selective inhibition of a specific HIF-α isoform in the context of cancer is an attractive proposal because it would allow enhanced specificity and targeting of therapy. Appropriate focus on the development of small molecule inhibitors of SINHCAF would provide an important therapy to reverse the effects of this protein whose deregulation is implicated in multiple disease processes. It might also be useful in predicting which patients might respond to HDAC inhibitors, which are already developed but have received conflicting results in clinical trials [80].

## Abbreviations

AcH3: acetylated histone H3
ChIP: chromatin immunoprecipitation
HDAC: histone deacetylase
HIF: hypoxia-inducible factor
NF-κB: nuclear factor kappa B
ODD: oxygen-dependent degradation
PARP: poly-ADP ribose polymerase
PHDs: prolyl hydroxylases
SINHCAF: SIN3A and HDAC-associated factor
siRNA: small interfering RNA
SP1: specificity protein 1
TSA: trichostatin A
vHL: von Hippel– Lindau
