# Supplementary material for: SINHCAF/FAM60A and SIN3A specifically repress HIF-2α expression
Source: Biochem J. 2018 Jun 29;475(12):2073–90. doi: 10.1042/BCJ20170945 (PMC6024822; doi:10.1042/BCJ20170945)
Supplement: Supplementary Figures [file BCJ-475-2073-s1.pdf]

Supplemental Figure Legends Biddlestone et al.

SINHCAF/FAM60A and SIN3A specifically repress HIF 2α expression

Figure S1: SINHCAF as a HIF 2α repressor. (A) qPCR validation using HeLa cells, for SINHCAF and SIN3A. Graphs depict mean + S.E.M. * $P \leq 0.05$, ** $P \leq 0.01$, *** $P \leq 0.001$. (B) Quantification of HIF 2α protein levels following SINHCAF depletion compared to control cells exposed to hypoxia. Graph depicts mean and S.E.M. of HIF 2α protein levels normalised to actin of a minimum of 3 independent experiments. Graphs depict mean + S.E.M. * $P \leq 0.05$, ** $P \leq 0.01$, *** $P \leq 0.001$. (C) Quantification of HIF 2α protein levels following SIN3A depletion compared to control cells exposed to hypoxia. Graph depicts mean and S.E.M. of HIF 2α protein levels normalised to actin of a minimum of 3 independent experiments. Graphs depict mean + S.E.M. * $P \leq 0.05$, ** $P \leq 0.01$, *** $P \leq 0.001$. (D) Quantification of HIF 2α protein levels following SINHCAF, PHD2 or SINHCAF and PHD2 depletion compared to control cells exposed to hypoxia. Graph depicts mean and S.E.M. of HIF 2α protein levels normalised to actin of a minimum of 3 independent experiments. Graphs depict mean + S.E.M. * $P \leq 0.05$, ** $P \leq 0.01$.

Figure S2: **SINHCAF represses the HIF 2α promoter.** (A) U2OS-HIF 2α promoter cells were transfected with 1μg of control of SINHCAF expressing plasmids prior to lysis and luciferase assay being performed. Graphs depict mean + S.E.M. * $P \leq 0.05$, ** $P \leq 0.01$, *** $P \leq 0.001$.

Figure S3: Validation of SP1 and SP3 knockdown levels. (A) qPCR and western blot validation for siRNA using HeLa cells. Graphs depict mean + S.E.M. * $P \leq 0.05$, ** $P \leq 0.01$, *** $P \leq 0.001$. HeLa cells were transfected with the indicated siRNA oligonucleotides and exposed where indicated to 1% O2 for 24 hours prior to cell lysis. Whole cell lysates were analysed by immunoblot for the levels of the depicted proteins.

Figure S4: Colony formation assay following SINHCAF and HIF 2α. (A) Colony Formation: HeLa and DLD-1 cells were seeded for colony formation following siRNA transfection. Representative images are displayed.

Sup. Figure S1

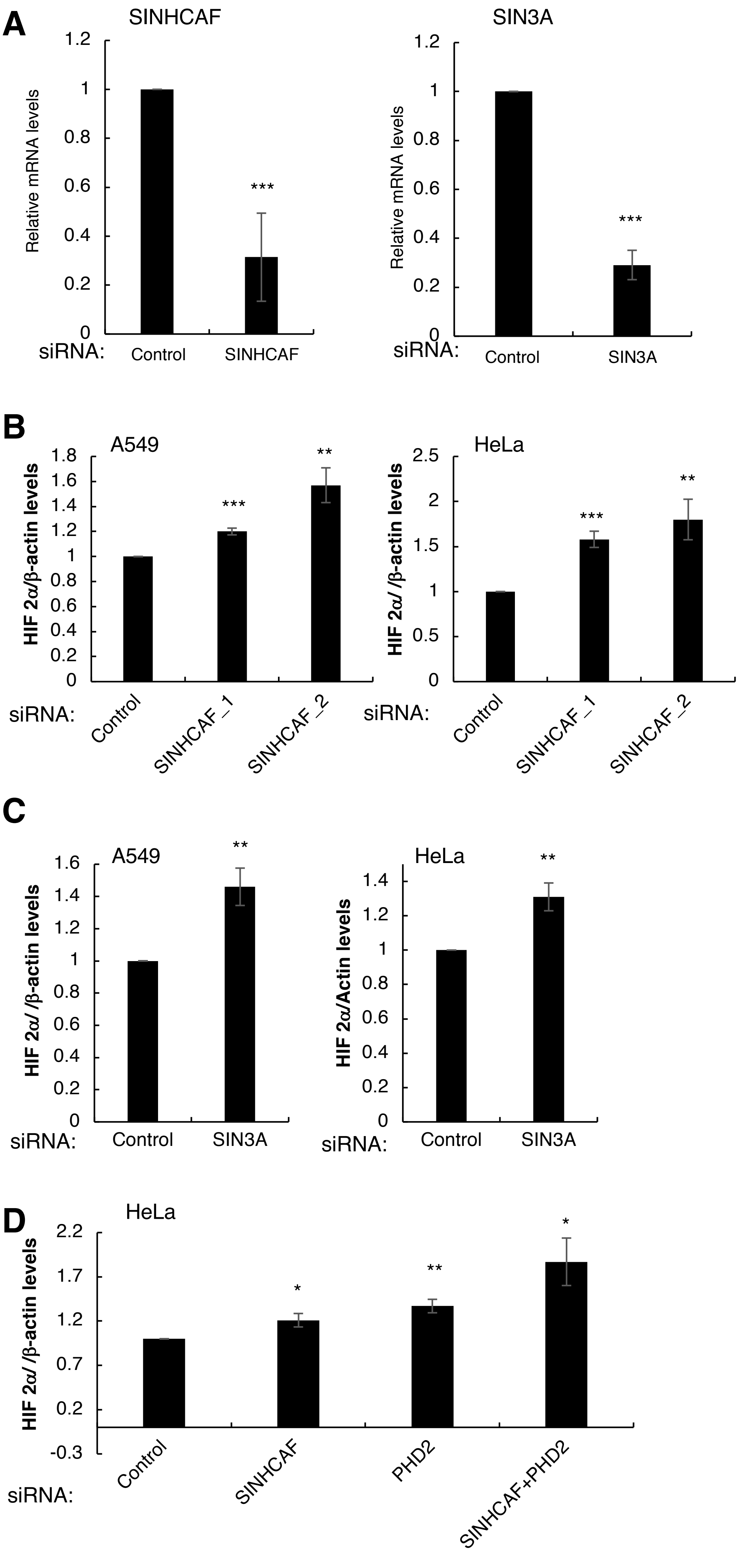

Sup. Figure S2

A

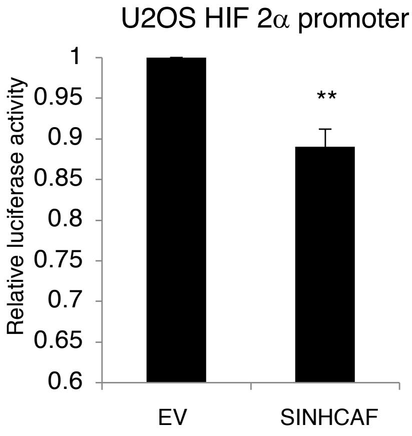

Sup. Figure S3

A

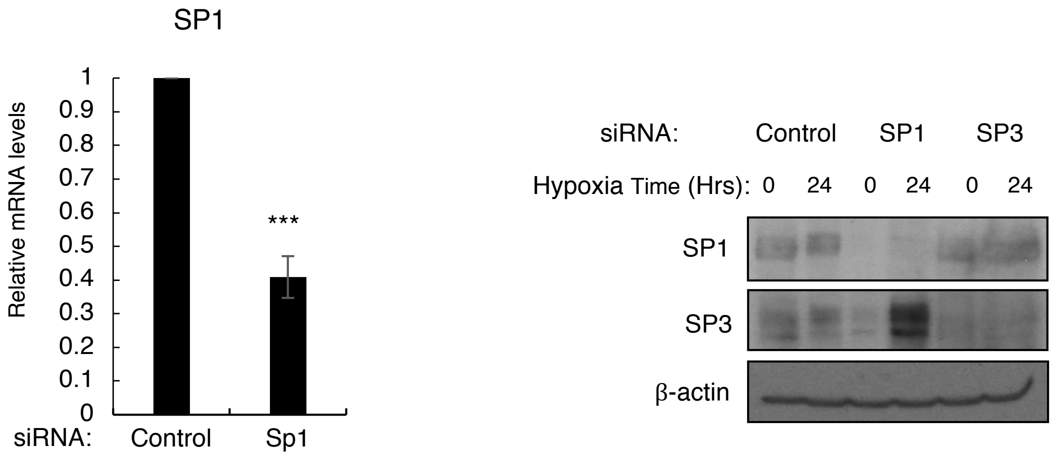

Sup. Figure S4

A

Control SINHCAF_2 SINHCAF_1 +HIF 2α

HeLa

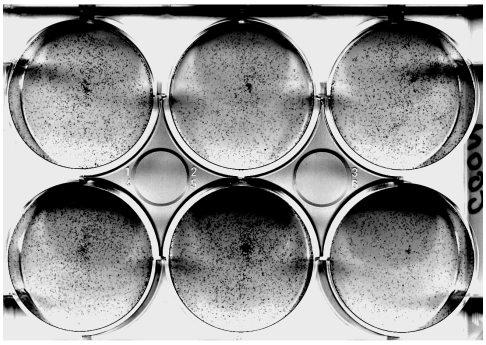

SINHCAF_1 HIF 2α SINHCAF_2 +HIF 2α

Control SINHCAF_2 SINHCAF_1 +HIF 2α

DLD-1

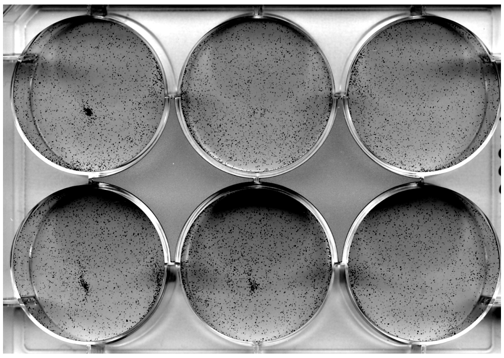

SINHCAF_1 HIF 2α SINHCAF_2 +HIF 2α
